# An organic extract from ascidian *Ciona robusta* induces cytotoxic autophagy in human malignant cell lines

**DOI:** 10.3389/fchem.2024.1322558

**Published:** 2024-02-08

**Authors:** Alessandra Gallo, Ylenia Maria Penna, Maria Russo, Marco Rosapane, Elisabetta Tosti, Gian Luigi Russo

**Affiliations:** ^1^ Department of Biology and Evolution of Marine Organisms, Stazione Zoologica Anton Dohrn, Naples, Italy; ^2^ National Research Council, Institute of Food Sciences, Avellino, Italy

**Keywords:** marine organisms, ascidians, *Ciona robusta*, cytotoxicity, autophagy, cell viability, anticancer agents

## Abstract

The last decades have seen an increase in the isolation and characterization of anticancer compounds derived from marine organisms, especially invertebrates, and their use in clinical trials. In this regard, ascidians, which are included in the subphylum Tunicata, represent successful examples with two drugs, Aplidine^©^ and Yondelis^©^ that reached the market as orphan drugs against several malignancies. Here, we report that an organic extract prepared from homogenized tissues of the Mediterranean ascidian *Ciona robusta* inhibited cell proliferation in HT-29, HepG2, and U2OS human cells with the former being the most sensitive to the extract (EC_50_ = 250 μg/mL). We demonstrated that the ascidian organic extract was not cytotoxic on HT-29 cells that were induced to differentiate with sodium butyrate, suggesting a preference for the mixture for the malignant phenotype. Finally, we report that cell death induced by the organic extract was mediated by the activation of a process of cytotoxic autophagy as a result of the increased expression of the LC3-II marker and number of autophagic vacuoles, which almost doubled in the treated HT-29 cells. In summary, although the detailed chemical composition of the *Ciona robusta* extract is still undetermined, our data suggest the presence of bioactive compounds possessing anticancer activity.

## 1 Introduction

The last decades have seen increased isolation and characterization of anticancer compounds derived from marine organisms and their use in clinical trials. The initial studies in this field, dating back to the 70s of the last century, originated from the interest of naturalists and marine biologists, who identified various toxins present only in the organisms that inhabit marine ecosystems. Examples are Conidia, a family of marine gastropod mollusks that inject a potent peptide toxin (conotoxin) to immobilize their prey (McManus et al., 1981); the cnidarians Zoanthids, commonly found in coral reefs, possessing a toxic polyketide “palytoxin,” which makes them unpleasant to predators (Moore and Scheuer, 1971); some microalgae produce a highly cytotoxic alkaloid neurotoxin, saxitoxin, and a polyketide neurotoxin, brevetoxin (Simmons et al., 2005). In recent years, studies to isolate new substances with anticancer activity have intensified thanks to a series of research programs supported by various institutions, such as the National Cooperative Drug Discovery Program of the National Cancer Institute (NCI). Subsequently, the development of collaborations between groups of academic researchers and major pharmaceutical companies, such as the NCI, has led to the development of several agents that have entered clinical trials, which have been excellently reviewed in recent works (Sorokina and Steinbeck, 2020; Cooreman et al., 2023). In these studies, interesting links can be found to updated databases on natural products such as those of marine origin.

Although the main marine sources of bioactive compounds are represented by invertebrates, largely sponges, tunicates, and soft corals, the observation that microorganisms such as marine microalgae, cyanobacteria, and heterotrophic bacteria live in symbiosis with them has led in the late 90s to formulate the “symbiotic origin” hypothesis for some of the most bioactive and druggable natural products (Haygood et al., 1999; Piel, 2004; Piel, 2004; Piel, 2009; Piel, 2009). Metagenomics analyses have validated this concept, providing insights into the possibility of culturing marine microorganisms as resources for new classes of therapeutic drugs (Williams, 2009; Cooreman et al., 2023).

Successful examples come from the marine species of the class Ascidiacea included in the subphylum Tunicata of the phylum Chordata. Ascidiacea, commonly known as ascidians or sea squirts, are grouped into three orders: Aplousobranchia, Phlebobranchia, and Stolidobranchia. The genomes of 20 ascidian species are deposited in the National Center for Biotechnology Information (NCBI) database^1^. Plitidepsin (dehydrodidemnin B; Aplidine^©^) is a cyclic depsipeptide with anticancer properties that was originally isolated from the Caribbean tunicate *Trididemnum* sp. (Rinehart et al., 1981). Subsequently, the same compound has been isolated from the marine α-proteobacterium *Tistrella mobilis*, hosted by the same tunicate (Tsukimoto et al., 2011). Plitidepsin, under the commercial name of Aplidine^©^, has been approved as an orphan drug for several hematological malignancies, such as acute lymphoblastic leukemia, multiple myeloma, primary myelofibrosis, and post-essential thrombocythemia myelofibrosis (Alonso-Álvarez et al., 2017; Cooreman et al., 2023). More recently, promising preclinical effects have been demonstrated for Aplidine^©^ in treating SARS-CoV-2 infection in two different mouse models, suggesting potential therapeutic applications against COVID-19 (White et al., 2021).

More than 35 years have passed since the discovery of another bioactive molecule, which was originally listed among the most promising antibiotic anticancer drugs derived from marine organisms, Ecteinascidin-743 (also named ET-743, trabectedin, Yondelis^©^) (Rinehart et al., 1990). ET-743 was isolated from the tunicate *Ecteinascidia turbinata* and later identified as a product of the bacterial symbiont *Candidatus* Endoecteinascidia frumentensis (Rath et al., 2011). The mechanism of ET-743 is unique in that it binds to the DNA minor groove and alkylates guanine in the N2 position preferentially after a GG or GC sequence. These ET-743-DNA adducts are recognized by the NER (DNA—nucleotide excision repair) system but unlike other adducts caused by alkylating agents such as cisplatin, the NER system causes not only DNA repair but also cell death even at very low concentrations (10–100 ng/mL) and preferentially via the mitochondrial pathway, caspase-3, and JNK kinase activation (Takebayashi et al., 2001; D'Incalci et al., 2002; D'Incalci and Galmarini, 2010). The clinical story of ET-743 is partially controversial. Between 2001 and 2003, under the name of Yondelis^©^, the drug received the status of orphan designation by EMA in the European Union for the treatment of soft tissue sarcoma and ovarian cancer. In 2015, the FDA approved it for treating soft tissue sarcoma, such as unresectable liposarcoma and leiomyosarcoma in metastatic patients. However, the conclusions reached by several trials on the toxicity and efficacy/safety balance of Yondelis^©^ led the EMA and FDA agencies to re-evaluate the marketing authorization of Yondelis^©^ (reviewed in Cooreman et al., 2023). Despite the examples of the symbiotic origin of plitidepsin and trabectedin, the synthetic source of the large part of active metabolites isolated from ascidians remains unknown (Tianero et al., 2015) and cannot be excluded in that some of them may have been derived from unidentified dietary sources.

Considering the success of ascidian-derived agents in clinical trials, several years ago, searching for alternative sources of anticancer agents, we demonstrated that a methanolic extract prepared from the tissues of the ascidian *Ciona intestinalis* (class: Ascidiacea; order: Phlebobranchia; family: Cionidae) possessed antiproliferative and pro-apoptotic effects on cancer cell lines of different origin (Russo et al., 2008). *Ciona intestinalis* represented an ideal candidate since its abundant presence in the Mediterranean Sea, its genome was deposited (De Luca Di Roseto et al., 2002; Satoh et al., 2003; Shi et al., 2005), and its anatomy, physiology, and development were well known (Passamaneck and Di Gregorio, 2005; Tosti et al., 2011). However, it is important to clarify that the mentioned work on the methanolic extract from *C. intestinalis* (Russo et al., 2008) has been *bona fide* obtained from the species *Ciona robusta*, not *C. intestinalis*. In fact, until 2015, *C. robusta* and *C. intestinalis* were formerly considered the same organism, being almost indistinguishable. Recent morphological, ecological, and genomic data reached the conclusion that the populations of *C. intestinalis* from the Mediterranean Sea, the Pacific Ocean (Australia, Japan, New Zealand, South Korea, and west coast of North America) and the Atlantic coasts of South Africa (formerly known as *C. intestinalis* type A specimen) were similar to each other but differed from the type B specimen of *C. intestinalis* present on the East Coast of North America, the coast of Northern Europe, and the Bohai Sea and Yellow Sea. Based on the morphological and genetic data, it has been recently established that *C. intestinalis* type A and *C. robusta* correspond (Caputi et al., 2007; Brunetti et al., 2015).

In the present work, we establish a new protocol to isolate an organic fraction from homogenized tissues of *C. robusta* possessing antiproliferative activity on malignant cell lines. The bioactive extract could activate the process of cytotoxic autophagy.

## 2 Materials and methods

### 2.1 Chemicals

Crystal violet, formalin, chloroquine, sodium butyrate (NaBt), dimethyl-sulfoxide (DMSO), p-nitrophenyl phosphate (pNPP), and trypan blue were purchased from Merck/Sigma (Milan, Italy); gentamicin and kanamycin were purchased from PanReac AppliChem (Darmstadt, Germany); phosphate-buffered saline (PBS) tablets were purchased from Euroclone (Milan, Italy). All other chemicals used were of the highest research purity grade.

### 2.2 Preparation of bioactive extracts from ascidian *Ciona robusta*

This study did not include endangered or protected ascidian species and was conducted according to the guidelines of the Declaration of Helsinki amended by the European Directive 2010/63 on the protection of animals used for scientific purposes, transposed into the Italian law by Legislative Decree (2014)/26.

Bioactive extracts from *C. robusta* were prepared by combining different protocols previously published (Sakai et al., 1992; Russo et al., 2008) and summarized in the scheme reported in Figure 2. Briefly, adults of *C. robusta* were collected from different marine sites in the Mediterranean Sea (Gulf of Naples and Gulf of Taranto, Italy), which are not privately owned or protected in any way, according to the Italian legislation (DPR 1639/68, 19 September 1980, confirmed on 10 January 2000). After collection, ascidians were transported to the Marine Biological Resources service of Stazione Zoologica Anton Dohrn and acclimated for at least 7 days before use in tanks (1 animal/L) with running natural seawater (temperature of 18°C ± 2°C, pH 8.1 ± 0.1, salinity 39 ± 0.5 psu) equipped with oxygen pumps and fed daily with the Shellfish Diet 1800.

Small groups of samples (3–4 individuals depending on their length ranging between 2 and 5 cm) were collected, their external tunic removed, and the seawater in excess eliminated by gently squeezing. The remaining tissues were transferred in a 50 mL plastic tube, chopped into small pieces with scissors to facilitate homogenization and added to approximately three volumes (w/v) of isopropyl alcohol (*i*-PrOH). The crude homogenate obtained after homogenization using an Ultra-Turrax T25 blender (20,000 rpm; 5 min) was centrifuged at 3,000×*g* for 10 min. The *i*-PrOH extract was concentrated by using a rotovapor to obtain an aqueous emulsion, which was extracted thrice in an equal volume of ethyl acetate using a pear-shaped separatory funnel. The three organic, upper phases (OrPh) were combined, dried by using a rotovapor, and then redissolved in MeOH. The material soluble in MeOH was quantitated by weight, and a stock solution of 25 mg/mL was prepared for the subsequent cytotoxicity assays. An aliquot of the lower aqueous phase (AqPh), which contained approximately 90% in weight of the total material present in the aqueous emulsion after the *i*-PrOH extraction, was brought to a stock solution of 200 mg/mL to be applied to cell cultures. Both the organic and aqueous phases were stored at −20°C.

For the experiments in the presence of antibiotics, 15 individuals of *C. robusta* were reared for 3 weeks in a closed system tank containing natural seawater and a mixture of gentamicin and kanamycin (100 mg/L) under the same condition of acclimatization. The seawater was changed, and antibiotic solutions were prepared daily. Control individuals were maintained in identical experimental conditions avoiding the addition of antibiotics.

### 2.3 Cell culture and reagents

The HT-29 cell line, derived from a 44-year-old Caucasian patient suffering from colorectal adenocarcinoma (Von Kleist et al., 1975), was purchased from the American Tissue Culture Collection (ATCC) and LGC Standards (Sesto San Giovanni, Milan, Italy). The HepG2 cell line, isolated from a hepatocellular carcinoma of a 15-year-old male youth with liver cancer (Aden et al., 1979), and the U2OS cell line, derived from a moderately differentiated sarcoma of the tibia of a 15-year-old female osteosarcoma patient (Ponten and Saksela, 1967), were kindly donated by Professor M.A. Belisario from the University of Salerno (Salerno, Italy) and Professor A. Oliva from the Luigi Vanvitelli University (Naples, Italy), respectively.

The cell lines were cultured at 37°C in a humidified atmosphere with 5% CO_2_ using Dulbecco’s Modified Eagle’s Medium (DMEM; Euroclone, Pero, Milan, Italy) supplemented with 10% fetal bovine serum (FBS; Euroclone) with 100 μg/mL penicillin/streptomycin, 2 mM L glutamine, and 100 µM non-essential amino acids (Euroclone). To avoid any variations caused by the long-term culture of the cells, we used early passages (<10) to ensure reproducibility. Cell propagation was carried out by treating them with trypsin/EDTA solution (Euroclone) until confluence and counting/replating using trypan blue exclusion dye in an automatic cell counter (EveTM, NanoEnTek distributed by VWR, Milan, Italy) in accordance with the manufacturer’s instructions.

Treatments with the *C. robusta* extracts were performed at the times and concentrations indicated in the Results and Discussion section and in the figure legends. At the end of the experiments, the cells were analyzed to measure changes in cell viability, autophagy, and expression of autophagy markers as reported below. The cells were plated at an appropriate density in quadruplicate in a 96-well plate and allowed to adhere for 24 h before performing the functional assays.

Differentiation of HT-29 cells was obtained by treatment for 0–120 h with 4 mM NaBt as described (Russo et al., 1997; Russo et al., 1999) before the addition of the *C. robusta* extracts.

We measured the formation of H_2_O_2_ by using the ferrous oxidation–xylenol orange (FOX) assay as reported (DeLong et al., 2002) after incubation of *C. robusta* extracts at the concentrations indicated in the Results and Discussion section with the cell culture medium in the absence of cells. The FOX assay is based on the oxidation of ferrous (II) ion to ferric (III) ion in an acid environment. The molecular complex formed by the ferrous ion (II) and the relative dye is determined calorimetrically. A specific application of the FOX method is to identify oxidant species produced in the cell culture medium by the pro-oxidant activity of antioxidant molecules or extracts in the presence of high concentrations of metal ions (Fenton reaction).

### 2.4 Cell viability assays

The cell viability assay was performed primarily using the crystal violet technique, which uses a protein-binding dye (Tedesco et al., 2013; Feoktistova et al., 2016). Briefly, to eliminate any residue of the culture medium, before staining, a washing was carried out for 15 min with the PBS solution followed by the addition of 10% formalin in PBS to allow fixation. Finally, crystal violet 0.02% (w/v) in an aqueous solution was incubated for 30 min before dye removal and observation of the stained cells in a bright-field invertoscope (Axiovert 200, ZEISS) with 200–400× magnification to allow high-resolution photographs to be taken. Quantification was carried out by adding a 10% (v/v) acetic acid solution to solubilize the dye incorporated within the cells followed by a spectrophotometric reading at a wavelength of 595 nm using a microplate reader (Synergy HT BioTek, Milan, Italy).

The CyQUANT (Thermo Fisher Scientific/Life Technologies, Milan, Italy) method was performed essentially as previously described (Russo et al., 2017b). In the presence of the reagent called “background suppressor” that penetrates through the pores that form on the membranes of damaged cells, the CyQUANT dye could no longer intercalate into the nuclear DNA and dead cells did not fluoresce. Briefly, the CyQUANT mixture was added to the cells following incubation for 1 h at 37°C following the manufacturer’s instructions. The CyQUANT kit contains the nuclear dye (CyQUANT nuclear stain; 1:250 dilution) and suppressor of basal fluorescence (background suppressor; 1:50 dilution), which discriminates between living and dead cells and avoids staining the latter. Fluorescence was measured at the excitation wavelength of 485 nm and emission wavelength of 530 nm. The results were expressed as a percentage of fluorescence of the untreated control using a microplate reader (Synergy HT BioTek).

### 2.5 Measurement of autophagy

Autophagy was monitored in live cells using the Cyto-ID Autophagy detection kit (Enzo Life Sciences, Milan, Italy) (Russo et al., 2023). Autophagy activation was detected using this specific kit based on a fluorescent amphipathic cationic tracer that could specifically detect the number of intracellular autophagosomes but not the lysosomes. The cells (approximately 1 × 10^4^ cells per well) were plated in a 96-well plate (Corning Costar, Euroclone) and treated with the *C. robusta* extracts as indicated in the Results and Discussion section. After treatment, the medium was removed, and the cells were washed with the assay buffer containing 5% FBS. The mixture (100 µL) with the autophagy detection marker (Cyto-ID, diluted 1:500) and nuclear dye (Hoechst 33342, diluted 1:1000) was added to phenol red-free DMEM at 5% FBS and allowed to react for 30 min in the incubator at 37°C in 5% CO_2_ atmosphere. The cells were washed with the assay buffer before taking photographs and making a double fluorometric measurement using a microplate fluorescence reader (Synergy HT BioTek). The autophagosomes were quantified by normalizing green fluorescence (Cyto-ID) at an excitation wavelength of 495 nm and an emission wavelength of 519 nm, as for fluorescein isothiocyanate (FITC). The second measurement concerning the blue fluorescence (Hoechst dye 33342) was performed at the wavelengths of 358/461 nm as for 4′,6-diamidin-2-phenylindole (DAPI). The FITC/DAPI ratio obtained in fluorescence units allowed the quantification of cells with autophagosomes compared to the total number of cells. A fluorescence microscope invertoscope (ZEISS Axiovert 200; ×400 magnification) was used to observe and take photos of autophagic vesicles and the nuclei through the application of FITC and DAPI filters.

### 2.6 Polyacrylamide gel electrophoresis and immunoblotting

HT-29 cells (generally 1.5×10^6^) treated with *C. robusta* extracts were lysed using a lysis buffer containing protease and phosphatase inhibitors (50 mM Tris/HCl, pH 7.4; 150 mM NaCl; 5 mM ethylenediaminetetraacetic acid; 1% Nonidet P-40; 0.5 mM dithiotreitol; 1 mM Na_3_VO_4_; 40 mM NaF; 1 mM Na_4_P_2_O_7_; 7.4 mg/mL 4-p-nitrophenyl phosphate; 10% glycerol; 100 μg/mL phenylmethylsulfonyl fluoride, and the cocktail of protease and phosphatase inhibitors (Merck/Sigma)) (Russo et al., 2017a). After the protein concentration determination using the Bradford method (Bradford, 1976), the total lysates (20–30 µg/lane) were added to a solution to reduce the disulfide bridges (Reducing Agent 20×; Bio-Rad, Milan, Italy) and Laemmli Sample Buffer 4× (Bio-Rad) (100 mM Tris/HCl, pH 6.8; 4% SDS; 200 mM dithiothreitol; 20% glycerol; 0.2% bromophenol blue dye). The samples were first heated for 5 min at 95°C and then centrifuged at 11,000×*g* in microfuge before loading them on 4%–12% precast mini- or midi-gels (Thermo Fisher Scientific/Life Technologies). The running buffer contained MOPS [3-(N-morpholino) propanesulfonic acid], (50 mM MOPS, 50 mM Tris, 1% SDS, 1 mM EDTA, pH 7) for the separation of high-molecular-weight proteins, while MES buffer [2-(N-morpholino) ethanesulfonic acid] (50 mM MES, 50 mM Tris, 1% SDS, 1 mM EDTA, pH 7) was used for low-molecular-weight proteins. A constant voltage (200 V) was set for the electrophoretic chamber (Thermo Fisher Scientific/Life Technologies). At the end of electrophoresis, proteins were transferred from the gel to a 0.2 µm polyvinylidene fluoride (PVDF) membrane (Transfer pack; Bio-Rad) using the Trans-Blot Turbo system (Bio-Rad) applying a constant amperage (2.5 mA) for 7 min at room temperature. After transfer, gels were stained using Coomassie blue R-250 (Merck/Sigma) to detect the untransferred proteins, and the membranes were washed with 1× T-TBS (Tween-20 in Tris-buffered saline) (0.1% Tween-20; 25 mM Tris, pH 8; 137 mM NaCl; 2.69 mM KCl) for 5 min with shaking and incubated for 1 h with a blocking solution (3% bovine serum albumin, or non-fat dry milk 5% in T-TBS containing 0.02% NaN_3_). The following primary antibodies were diluted 1:1,000 in 3% BSA/T-TBS and incubated for approximately 16 h: anti-LC3-I/II (cat. # 12741S) from Cell Signaling Technologies (distributed by Euroclone); anti-α-Tubulin (cat. # T9026; Merck/Sigma). Following washing with T-TBS, the membranes were incubated for 2 h with a secondary antibody linked to horseradish peroxidase (diluted 1:20,000 in T-TBS). Immunoblots were developed using the ECL Prime Western blotting detection system kit (GE Healthcare, Milan, Italy). Band intensities were quantified and expressed as optical density on a Gel Doc 2000 Apparatus (Bio-Rad) and Multi-analyst software (Bio-Rad).

### 2.7 Statistical analysis

The results have been expressed as mean ± standard deviation (SD) based on the values obtained from independent experiments carried out in duplicate, triplicate, or quadruplicate. Differences between the two groups were analyzed using the Student’s t-test (Excel MS software 2016), and the significance was established as indicated in the figure legends.

## 3 Results and discussion

To verify the capacity of the *C. robusta* organic extract to affect cell viability in cancer cells, we selected three cell lines, namely, HT-29, HepG2, and U2OS derived from a human colorectal adenocarcinoma cell line, a hepatocellular carcinoma, and a moderately differentiated osteosarcoma, respectively. These cell lines were selected based on the following criteria: i) they grow adherent and have in common an epithelial-like morphology even deriving from different cancer types; ii) they are generally resistant to cell death induced by chemotherapeutic and/or pro-apoptotic drugs; iii) they represent examples of tumors responding to the anticancer activity of other ascidian-derived agents (Cooreman et al., 2023).

**FIGURE 1:**
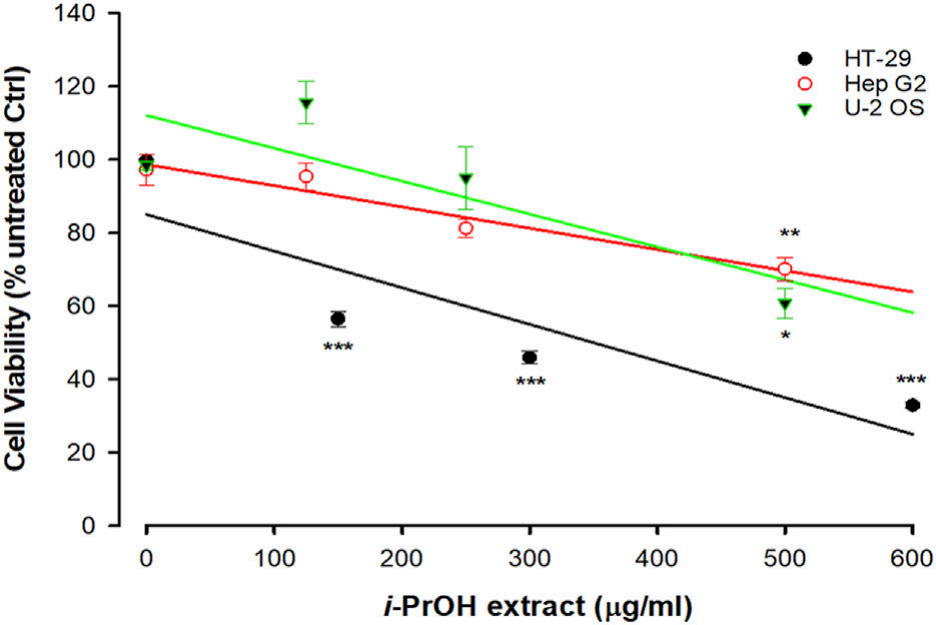
Cytotoxicity of *Ciona robusta* isopropyl extract on different cell lines. The cells (1 × 10^4^/well) were incubated with increasing concentrations of *i*-PrOH extract for 48 h. Cell viability was measured using the crystal violet assay, as reported in the Materials and Methods section. In the control (Ctrl) experiments (no *i*-PrOH extract), the cells were treated with vehicle (methyl alcohol 2% v/v, final concentration). Data are presented as the mean of three independent experiments ±SD. Symbols indicate significance: ***p < 0.0005; **p < 0.005; *p < 0.05 vs*.* untreated Ctrl (Student’s t-test).

**FIGURE 2:**
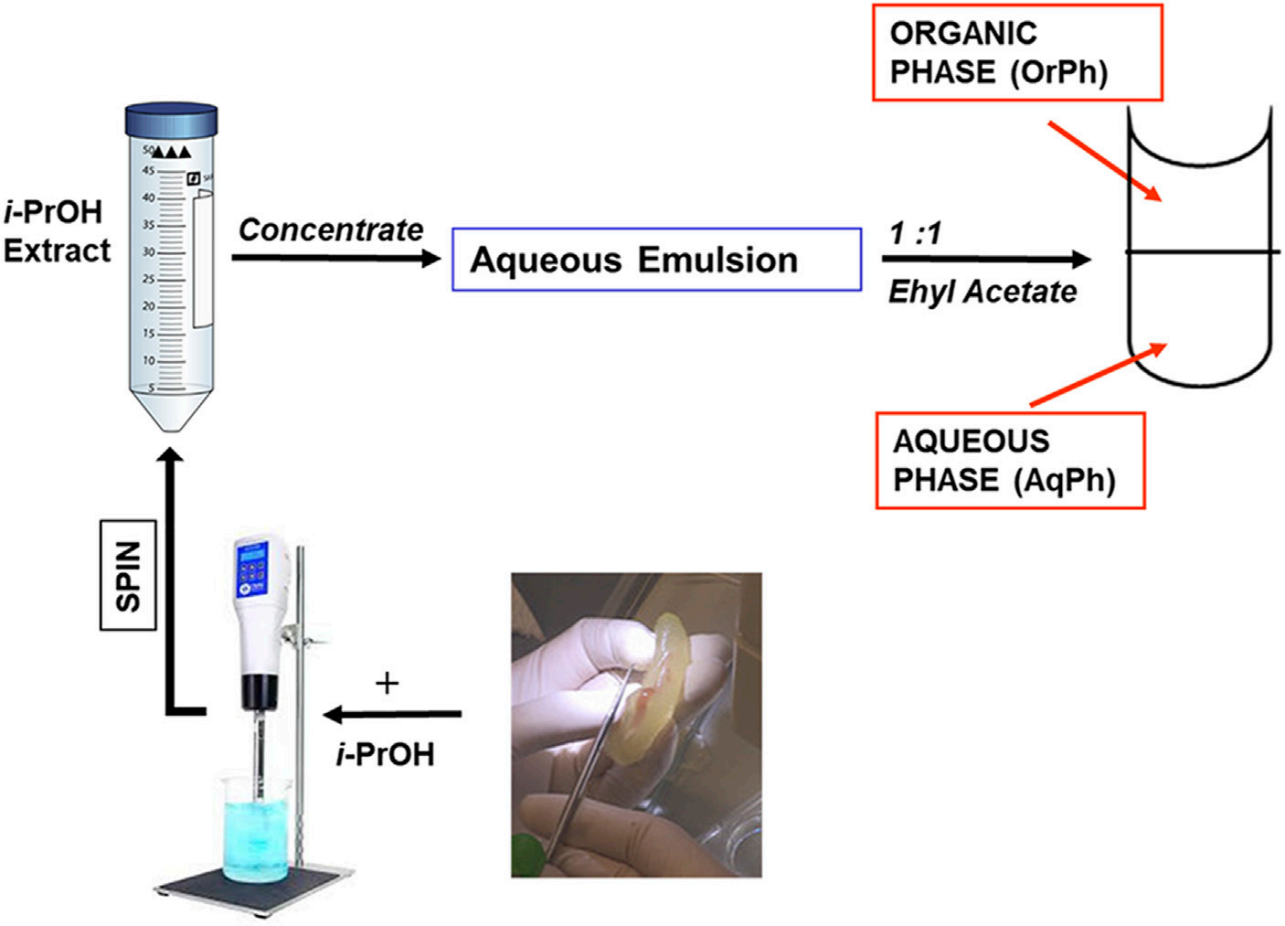
Preparation of the *Ciona robusta* extracts. The general scheme summarizes the different steps leading to the *C. robusta* extracts tested on the other cell lines reported in Figure 2. For details, see the description in the Materials and Methods section. *i*-PrOH, isopropyl alcohol; OrPh, organic phase; AqPh, aqueous phase.

As reported in Figure 1, the original isopropyl alcohol (*i*-PrOH) extract (Figure 2) showed a different grade of cytotoxicity on the selected cell lines, with HT-29 being the most sensitive with an EC_50_ of approximately 250 μg/mL when compared to HepG2 and U2OS (EC_50_ > 500 μg/mL).

Based on these results, HT-29 cells were selected as a leading cellular model for the next experiments. It is worthwhile to note that the screening of cell lines presented in Figure 1 extends upon the one previously reported (Russo et al., 2008) and indicates selective cytotoxicity of the ascidian extract on specific cancer types, with the human colorectal adenocarcinoma cell lines being among the most sensitive. In fact, HT-29 here and Caco-2 in our previous work (Russo et al., 2008) showed the lowest EC_50_.

**FIGURE 3:**
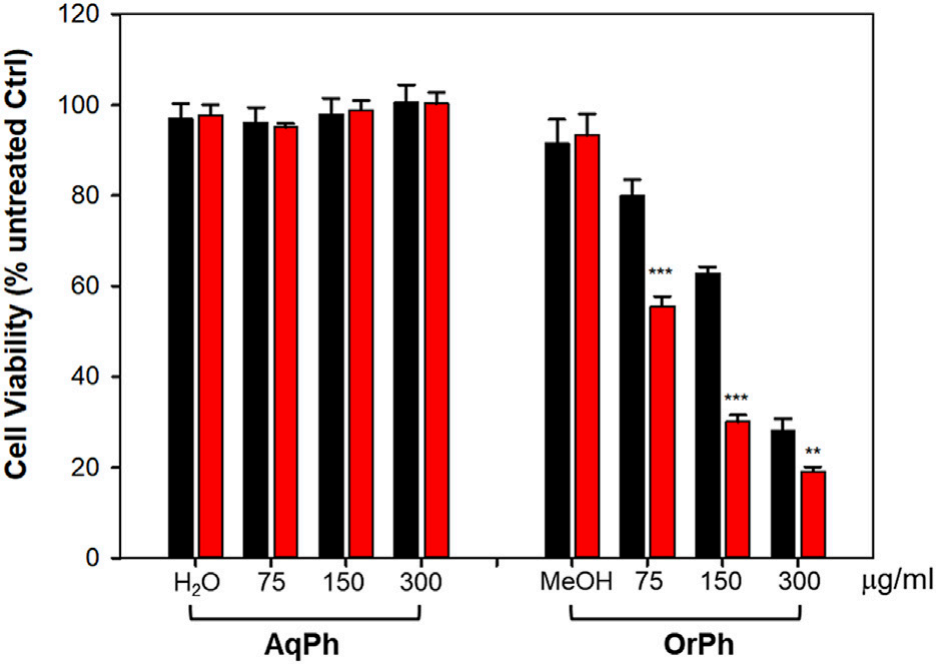
Cytotoxicity of *Ciona robusta* aqueous and organic extracts on HT-29 cell line. The cells (2 × 10^4^/well) were incubated with increasing concentrations (75–300 μg/mL w/v) of the aqueous (AqPh) and organic (OrPh) phases prepared as reported in the Materials and Methods section for 24 (black bars) and 48 h (red bars). Cell viability was measured using the crystal violet assay, as reported in the Materials and Methods section. As controls (Ctrl), water (H_2_O) and methyl alcohol (MeOH; 2% v/v, final concentration) were used in the AqPh and OrPh groups, respectively. Data are presented as the mean of three independent experiments ±SD. Symbols indicate significance: ***p < 0.0005; **p < 0.005 vs. untreated Ctrl (Student’s t-test).

The *i*-PrOH extract was further purified as indicated in Figure 2 and described in the Materials and Methods section to obtain two new phases, the aqueous (AqPh) and organic (OrPh) phases, whose effects on cell viability were measured on the HT-29 cell line. Figure 3 shows that the AqPh was not associated with any cytotoxic activity. On the contrary, the OrPh was enriched in the agents responsible for reducing cell viability as results from the time- and dose-dependent increase in cytotoxicity (Figure 3; right part of the graph). The calculated EC_50_ for the OrPh at 48 h resulted in approximately 90 μg/mL, thrice lower than the value reported in Figure 1 for HT-29 cells and referring to the crude *i*-PrOH extract.

**FIGURE 4:**
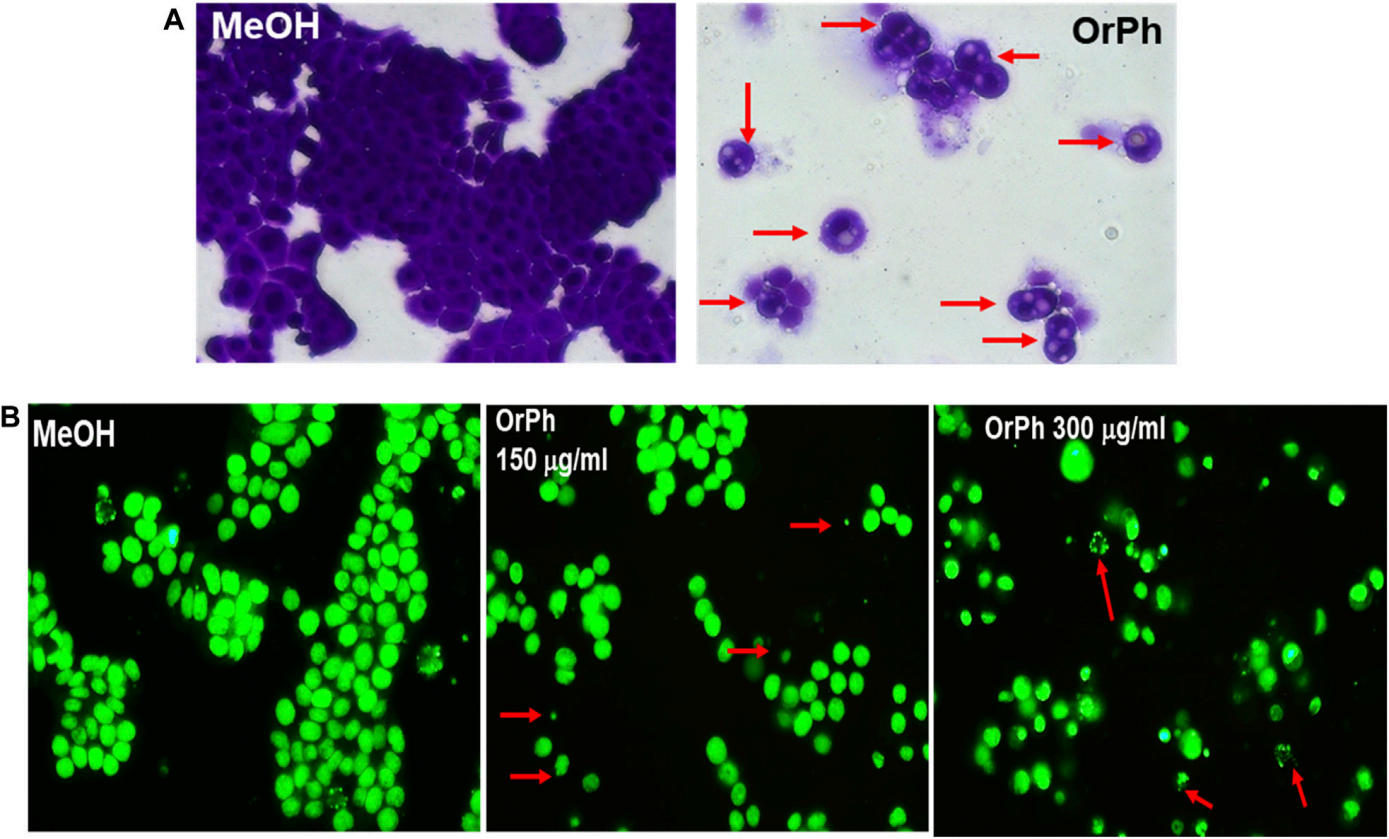
Cytotoxicity of *Ciona robusta* organic phase extract on the HT-29 cell line. Panel **(A)** Representative images (×400 magnification in bright field) of HT-29 cells treated as reported in the legend of Figure 3 and stained for 48 h with the crystal violet dye at the OrPh concentration of 300 μg/mL (w/v; right panel). The left panel indicates the treatment with the vehicle solvent (methyl alcohol; MeOH). Red arrows in the right panel indicate cells with vacuoles. Panel **(B)** Representative images of HT-29 cells treated as reported in panel **(A)** at the displayed concentration of OrPh (middle and right panels) or vehicle (MeOH; left panel) and stained with CyQUANT dye as reported in the Materials and Methods section to evidence the cell nuclei. Significant fields have been visualized with a ZEISS Axiovert ×200 fluorescence invertoscope and photographed with an FITC filter and ×400 magnification. Arrows in red indicate cells presenting apoptotic bodies.

The capacity of the OrPh fraction to induce cell death was corroborated by microscopy analyses (Figure 4). Figure 4A reports the morphology of HT-29 cells photographed at 48 h after treatment with the OrPh and subsequently fixed and stained with crystal violet. In its right panel, it is clear that the number of stained (and viable) cells is drastically reduced compared to the control experiment (left panel). Furthermore, the cytoplasm appears strongly vacuolated (red arrows). From the microscopy analysis, other information on the capacity of the OrPh fraction to induce the apoptotic cell death was obtained. In fact, the very sensitive CyQUANT fluorescence staining protocol allows the dye to enter the cells and intercalate into the DNA, emitting green fluorescence at 530 nm (FITC) in living cells. The result of this experiment is illustrated in Figure 4B, where the appearance of apoptotic bodies is clearly detectable (red arrows).

These data confirm the original observation that was previously reported by our group on a similar extract prepared from ascidian *C. intestinalis* (Russo et al., 2008) where the activation of apoptosis was demonstrated by the DNA ladder profile and by the activation of the caspase-3 proteolytic enzyme. However, in that study, the apoptotic effects were tested on leukemia cells growing in suspension, i.e., HL-60 (promyeloblasts isolated from acute promyelocytic leukemia) and HPB-ALL (established from the peripheral blood of acute lymphoblastic leukemia) cell lines, which, notoriously, are more sensitive to the cytotoxic action of naturally occurring agents/extracts than adherent cancer cells. In fact, we also measured a strong cytotoxic activity of the OrPh fraction on HL-60 and HPB-ALL cell lines (Russo and Gallo, 2024). However, here, we demonstrated that apoptosis is also triggered by the OrPh fraction in HT-29 cells, which are highly resistant to cell death induced by chemotherapy drugs, ionizing radiation (Russo et al., 2021), and apoptotic inducers, such as death receptors (Fas, TRAIL), and BH3 mimetic agents (Okumura et al., 2008; Ahmed et al., 2016; Nahacka et al., 2018).

One of the key questions associated with the antiproliferative capacity of new agents on cancer cells is the effects on normal cells to assess their specificity for the malignant phenotype and provide information on the risks vs. benefits ratio. The advantage of using the HT-29 cell line is represented by the capacity of these cells to differentiate when treated with differentiating agents such as sodium butyrate (NaBt) assessed by several biochemical and morphological markers. In fact, NaBt increases the activity of the alkaline phosphatase enzyme (AP), stimulates the appearance of mucin-producing cells and the formation of highly differentiated goblet-like enterocytes, and induces the expression of differentiation-associated cytokeratin proteins (Tanaka et al., 1990; Russo et al., 1997). These changes, mimicking the phenotype of normal intestinal epithelial cells, support the use of NaBt-differentiated HT-29 cells as a suitable model to compare the cytotoxicity of a given agent/extract in malignant cells vs*.* their differentiated and normal-like counterparts.

**FIGURE 5:**
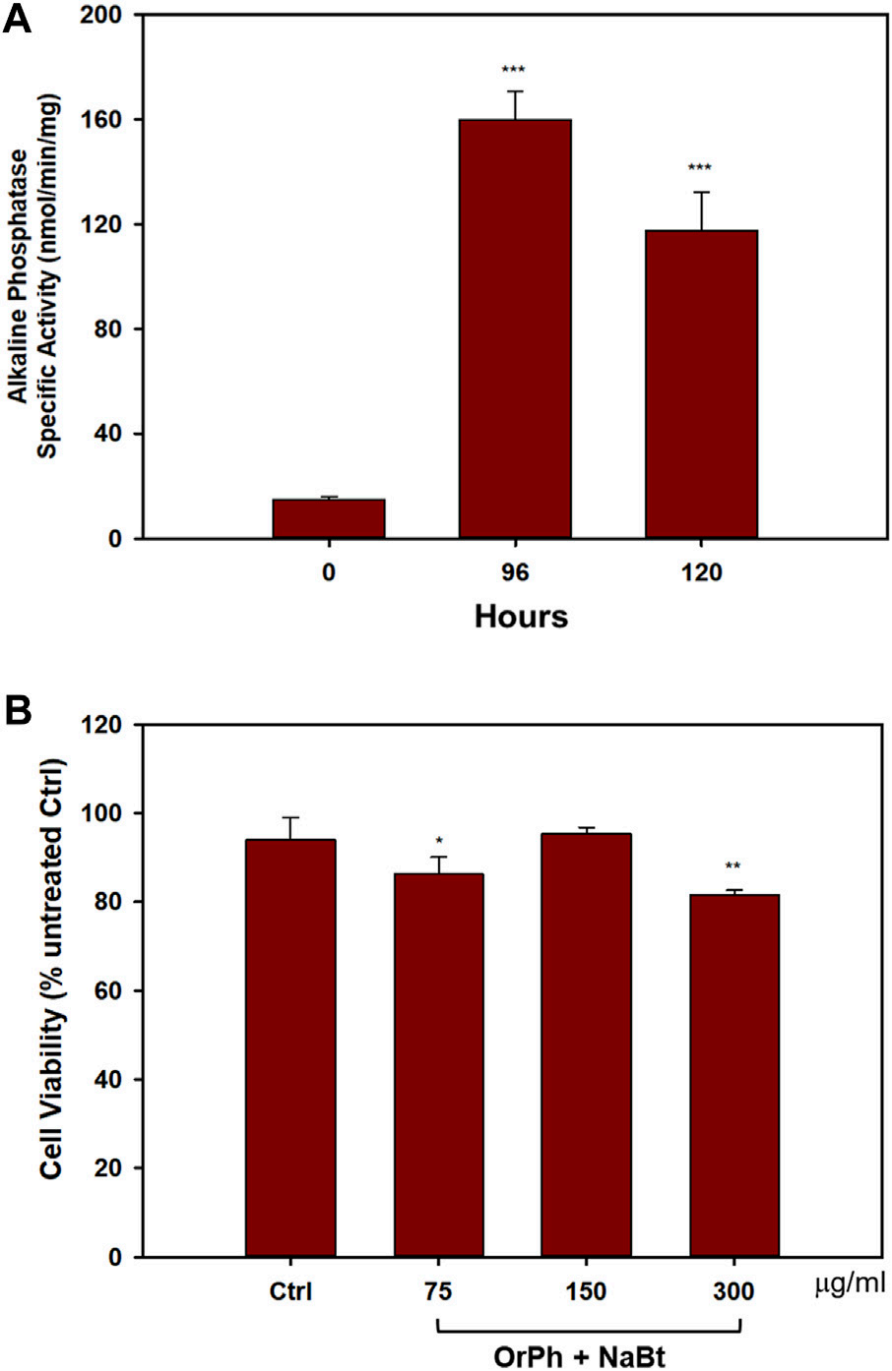
Effect of *Ciona robusta* organic phase extract on the differentiated HT-29 cell line. Panel **(A)** Cells (2 × 10^4^/well) were incubated with 4 mM NaBt for the indicated times, harvested, and lysed. The extracts were assayed to measure the activity of AP enzyme expressed as nanomoles of pNPP (p-nitrophenyl phosphate) per minute per milligram of total proteins assayed as reported in the Materials and Methods section. Data are presented as the mean of two independent experiments ±SD. Symbols indicate significance: ***p < 0.0005 vs. NaBt treated cells at time 0 (Student’s t-test). Panel **(B)** Cells treated with NaBt as described in panel **(A)** were added to the indicated concentrations of OrPh for 48 h. Cell viability was determined using the crystal violet assay, as reported in the Materials and Methods section. The control (Ctrl) point is represented by cells treated with MeOH (2% v/v). Data are presented as the mean of two independent experiments ±SD. Symbols indicate significance: **p < 0.005; *p < 0.05 vs*.* Ctrl (Student’s t-test).

On these premises, HT-29 cells were induced to differentiate with 4 mM NaBt, as demonstrated by the increase of approximately 9-fold of the AP activity (Figure 5A) between 96 and 120 h from the treatment. Differentiated cells were treated with increasing concentrations of OrPh fraction in the range of 75–300 mg/mL for 24 h. As reported in Figure 5B, the cytotoxic effect of the extract was minimal compared to undifferentiated HT-29 cells treated with identical concentration and time (Figure 3; bars on the right side of the graph).

This experiment is promising in supporting the hypothesis that the toxicity of the active components present in the *C. robusta* extract is specific for the malignant phenotype and does not interfere with the physiology of differentiated cells. It is important to remember that differentiated cells do not divide and are normally arrested in the G1 phase of the cell cycle. Therefore, massive cell death observed in undifferentiated HT-29 treated with the extract suggests that the bioactive compounds may trigger key modulators of the pathways regulating the G1/S transition or the G2/M phase of the cell division cycle.

Of course, data reported in Figure 5, although encouraging, should be considered only preliminary in assessing the absence and/or very limited cytotoxicity of *C. robusta* extract on normal cells. Screening other cell types is currently under scrutiny (e.g., human peripheral blood mononuclear cells from healthy donors and primary cells).

**FIGURE 6:**
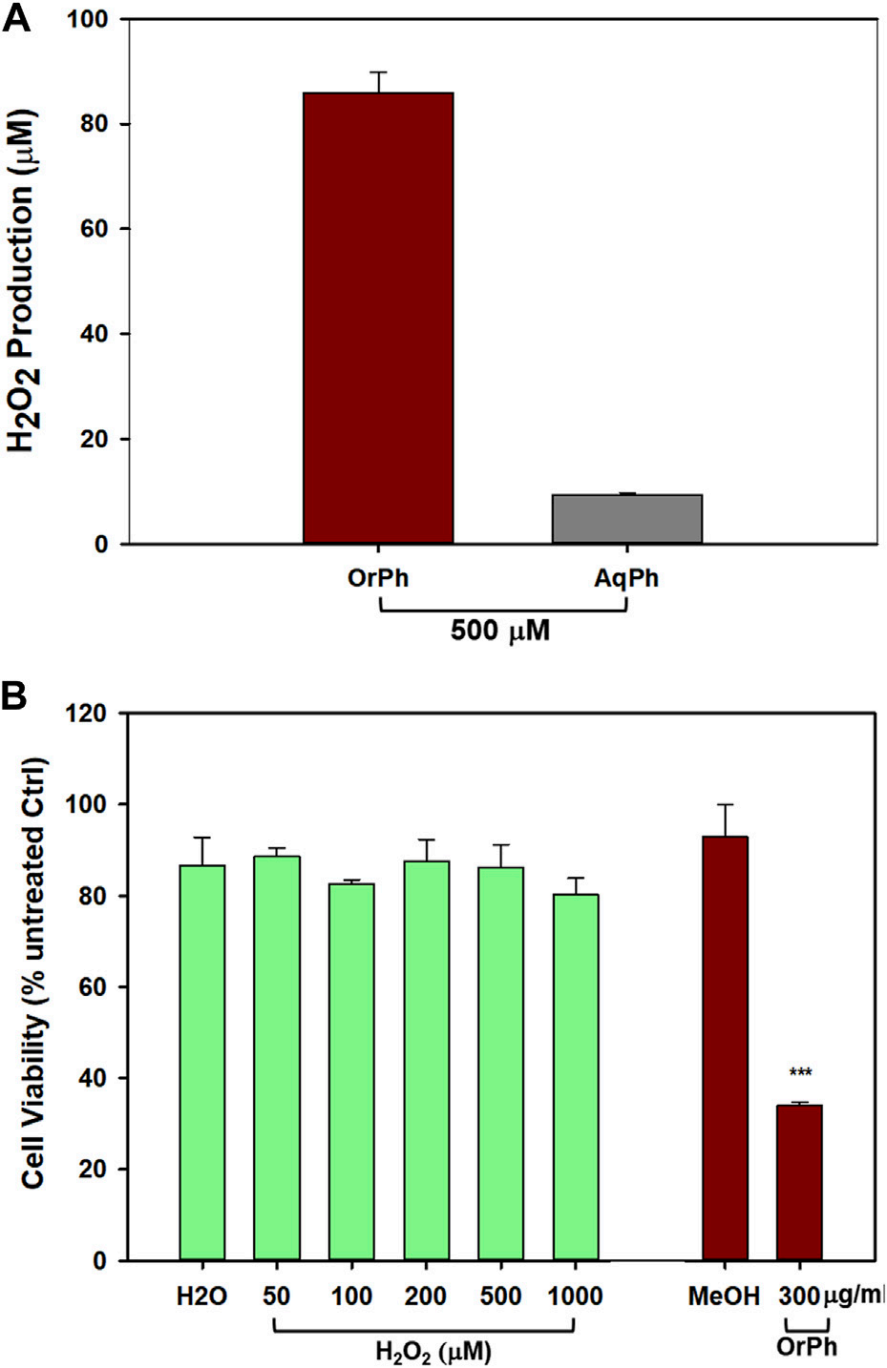
Effect of hydrogen peroxide production by *Ciona robusta* aqueous and organic extracts on the HT-29 cell line. Panel **(A)** The FOX assay was performed starting from the indicated concentration (500 μg/mL w/v) of both the aqueous (AqPh) and organic (OrPh) phase extracts in the absence of cells as reported in the Materials and Methods section. The production of H_2_O_2_ was measured after 24 h of incubation of the cell culture medium in the presence of the extracts. Panel **(B)** The cells (2 × 10^4^/well) were incubated with increasing concentrations (50–1,000 µM of H_2_O_2_ (green, left bars) or OrPh extract (300 μg/mL w/v) (dark right bars) for 24 h, and cell viability was measured using the crystal violet assay as reported in the Materials and Methods section. As controls, water (H_2_O) and MeOH (2% v/v) were used in the experiments, respectively. Data are presented as the mean of two independent experiments ±SD. Symbols indicate significance: ***p < 0.0005 vs*.* control (MeOH) (Student’s t-test).

When working with reactive extracts possessing biological activities, it is necessary to exclude artifactual phenomena due to the possibility that the observed cytotoxicity could be associated with the generation of hydrogen peroxide and/or other reactive oxygen species (ROS) following the interaction between the extracts and cell culture medium components. This possibility has been well described in the literature (Halliwell and Whiteman, 2004; Halliwell, 2009). To this aim, we first measured the formation of H_2_O_2_ by using the FOX assay after incubation of OrPh and AqPh extracts at the concentration of 500 μg/mL (w/v) with the cell culture medium in the absence of cells. The FOX method is widely used to measure levels of hydrogen peroxide and, more generally, hydroperoxides in biological systems. As an example, the method is used for the detection of levels of lipid hydroperoxides in plant, fruit, and vegetable tissues (DeLong et al., 2002). The results presented in Figure 6A indicate that while the OrPh fraction generated a significant amount of H_2_O_2_ (approximately 85 µM), a minimal production was observed for the AqPh fraction (approximately 10 µM). However, when we exposed HT-29 cells to increasing concentrations of H_2_O_2_ (up to 1,000 µM), no cytotoxicity was observed (Figure 6B; left side of the graph) compared to approximately 65% of cell death caused by the OrPh fraction alone (Figure 6B; right side of the graph).

The results presented in Figure 6 confirm the low response of HT-29 cells to oxidative insults, which justifies their high resistance to radio- and chemotherapy (Russo et al., 2021; Russo et al., 2023). However, and even more importantly, these data support the evidence that *C. robusta* OrPh extract contains agents that can interfere with the processes regulating cell growth and cell death in HT-29 that cannot be attributed to artifacts generated by the experimental conditions and are due to the direct interaction of such putative extract components with the malignant cells. This simple experiment is of crucial importance considering the role of ROS and oxidative damage in pathology (Forman and Zhang, 2021) and the importance of their correct determination in the different experimental settings, both in cells and *in vivo* (Murphy et al., 2022). In addition, in recent years, qualified literature and numerous research groups in the field suggest caution when interpreting data on the biological properties of molecules/extracts derived from naturally occurring sources since many of them may fall within the category known as pan-assay interference compounds (PAINS) that function as reactive chemicals rather than target-directed drugs (Russo, 2007; Baell and Walters, 2014). For example, PAINS are responsible for the unspecific production of hydrogen peroxide that causes interference with the redox regulatory mechanisms.

**FIGURE 7:**
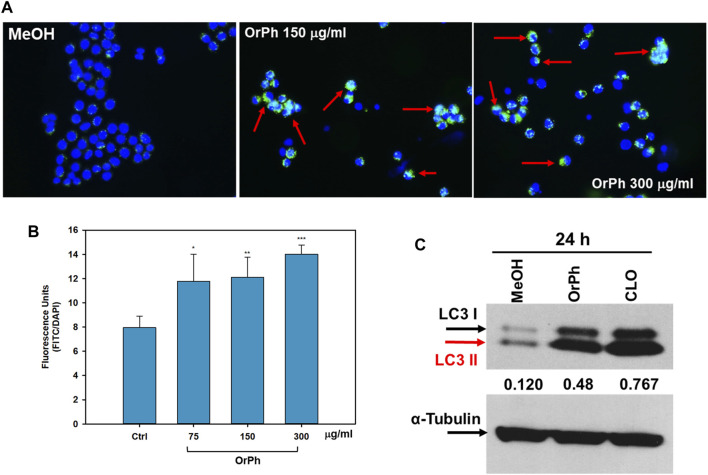
Activation of autophagy in HT-29 cells treated with *Ciona robusta* organic phase extracts. Panel **(A)** Representative images evidencing the autophagic vacuoles (in green) and the cell nuclei (in blue) using double staining with Cyto-ID/Hoechst. HT-29 cells were treated at the indicated concentrations of OrPh fraction (middle and right panels) or vehicle (left panel) for 48 h and stained with Cyto-ID/Hoechst, as reported in the Materials and Methods section. Significant fields have been visualized with a ZEISS Axiovert ×200 fluorescence invertoscope and photographed with an FITC filter and ×400 magnification. Red arrows indicate cells with autophagosomes. Panel **(B)** Fluorometric quantification of autophagosomes in HT-29 cells treated as reported in panel **(A)** with the indicated different concentrations of OrPh fraction. The bars indicate SD. The significance of the treatments compared to the vehicle solvent (Ctrl, methyl alcohol) was calculated with the Student’s test: *p < 0.05, **p < 0.005, ***p < 0.0005. **(C)** Immunoblotting to detect LC3-I/II in HT-29 cells treated with 300 mg/mL (w/v) of OrPh fraction. Chloroquine (20 μM, CLO) was used as the positive control. Densitometry values are indicated by numbers between the upper and lower panels and are calculated as the ratio between LC3-II (active isoform) and α-tubulin expressions.

From careful microscopy observation of data reported in Figure 4, it has emerged that HT-29 cells treated with the OrPh extract at the highest concentrations (300 μg/mL w/v) show the presence of vacuoles of different sizes in the cytoplasm or the fragmentation of the cytoplasm itself (Figure 4A). The nuclei stained with the CyQUANT fluorescent dye appeared normal in size until 48 h, and apoptotic bodies were detectable at 72 h (Figure 4B). This latter observation suggests that treatment with the OrPh extract could activate the phenomenon of cytotoxic autophagy or type II cell death accompanied by the onset of apoptosis at a later time (Doherty and Baehrecke, 2018; Russo and Russo, 2018). To confirm this hypothesis with quantitative measurements, the cells treated with different concentrations of *C. robusta* fractions for 48 h were stained with a selective fluorescent reagent (Cyto-ID assay) that could detect phagosomes, autophagosomes, and autolysosomes, which are formed in the different phases of autophagy. By contrast, the nuclei were stained with Hoechst fluorochrome 33342. As reported in Figure 7A, autophagy vacuoles characterized by green staining (FITC) were evidenced in the treated cells, while the nuclei emitted fluorescence at a blue wavelength (DAPI). The quantification of the vacuoles was determined by spectrofluorometric measurement of the FITC/DAPI ratio (Figure 7B). A significant increase was observed in the number of autophagic vacuoles compared to the control at not only the highest concentration (300 μg/mL) applied of the OrPh fraction but also at the lowest (75 μg/mL), where the level of cytotoxicity was not maximal.

To confirm the activation of the autophagic process, biochemical markers, such as the expression of LC3 isoforms, were employed. HT-29 cells were treated for 24 h with 300 μg/mL OrPh extract, and cell lysates were separated on SDS-PAGE to detect the expression of LC3-I/II by immunoblotting. The LC3 system mediates the formation of the autophagosome. It is present in two forms: the free cytosolic and inactive form (LC3-I) and the active form conjugated to phosphatidylethanolamine (LC3-II) (Klionsky et al., 2021). Figure 7C shows a fourfold increase in the lipidated form of LC3 (LC3-II) after treatment with the extract when compared to MeOH-treated control cells. The growth was comparable to that induced by chloroquine (CLO), used in the experiment as a positive control (Russo and Russo, 2018).

These results suggest that the OrPh extract prepared from *C. robusta* induces a change in the autophagy flux in HT-29 cells as early as 24 h after treatment. At longer times (48–72 h), a threshold is reached beyond which the autophagic process becomes lethal and cell death process is activated (Doherty and Baehrecke, 2018). It cannot be excluded that, alongside cytotoxic autophagy, necrosis and/or apoptosis may also occur, and future data (caspase activity measurement and apoptotic body measurement, assessment of Annexin V positivity) can confirm this hypothesis. In fact, our previous study (Russo et al., 2008) had demonstrated that a partially purified extract from *C. intestinalis* had a cytotoxic effect on various transformed cell lines, such as those of colon adenocarcinoma Caco-2, activating short-term apoptosis (caspase-3 activation at 12 h and nuclear DNA fragmentation at 24 h).

**FIGURE 8:**
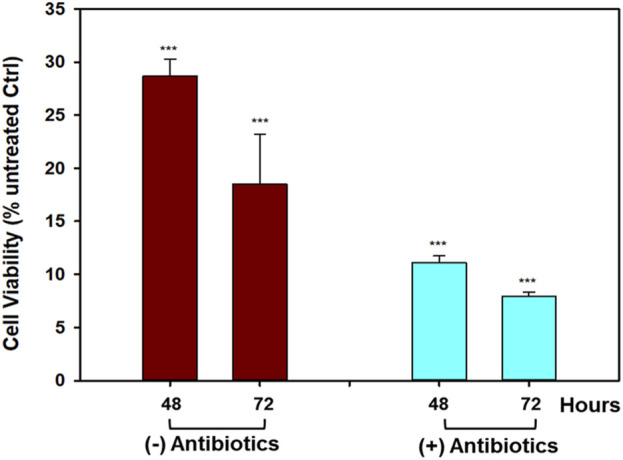
Effect of antibiotics treatment on the cytotoxicity induced by *Ciona robusta* organic extract on the HT-29 cell line. The cells (2 × 10^4^/well) were incubated with a concentration of OrPh phase corresponding to 300 μg/mL (w/v) for the indicated times and prepared from *C. robusta* colonies treated for 3 weeks with a cocktail of antibiotics (100 mg/L of gentamicin and kanamycin). After antibiotics incubation, the extracts were prepared as reported in the Materials and Methods section, and cell viability was measured using the crystal violet assay. Data are presented as the mean of two independent experiments ±SD. Symbols indicate significance: ***p < 0.0005 vs*.* cells treated with the vehicle solvent (MeOH 2% v/v; Student’s t-test).

To verify whether the antiproliferative activity of the OrPh extract prepared from *C. robusta* tissues was attributable to the presence of symbiotic microorganisms, colonies of *C. robusta* consisting of several dozen specimens were collected in the Gulf of Naples, maintained under aquaculture conditions, and treated with antibiotics (100 mg/L of gentamicin and kanamycin) for 3 weeks. Groups of control animals were kept under the same experimental conditions but in the absence of antibiotics. The OrPh extracts were prepared from antibiotics-treated and antibiotics-untreated samples and assayed on HT-29 cells under the aforementioned experimental conditions. The rationale of the experiment was to verify if, by removing the potentially antibiotics-sensitive microorganisms present in the adults of *C. robusta*, the cytotoxicity of the OrPh extract can be abolished. Data presented in Figure 8 clearly show that the presence of antibiotics does not modify the cytotoxic profile of the extracts, suggesting that the active compounds may not have a symbiotic origin. The reduced cell viability was even more pronounced in the antibiotics group than in the extract from control individuals. However, this difference may be due to the internal variability in the preparation procedures. It is essential to underline the expected behavior of the two OrPh extracts that were not influenced by the extensive treatment (up to 3 weeks) with a mixture of antibiotics. It is worthwhile to mention that maintaining individuals under microbiologically sterile conditions under the constant presence of a cocktail of antibiotics does not cause any apparent functional or physiological alteration. As an example, *in vitro* fertilization tests did not differ between the two groups concerning larval development.

The role of symbionts in assessing the presence of bioactive compounds with potential pharmacological properties in ascidians has been largely debated and recently extensively reviewed (Watters, 2018; Cooreman et al., 2023). It is well-established that the richness of the microbial diversity associated with ascidians (reviewed in Cooreman et al., 2023) and *C. intestinalis* represents a good example with approximately 33 different microbial genera, dominated by the Gammaproteobacteria class, in the gut microbiota (Utermann et al., 2020). However, if from one side, the structural resemblances existing between natural products isolated from marine organisms and metabolites produced by microorganisms suggest a common biosynthetic origin thus supporting the symbiotic theory, it is also known that the actual synthetic origin of the majority of metabolites in ascidians and other marine organisms remains undetermined (Tianero et al., 2015).

Data presented in Figure 8 are in favor of the non–symbiotic origin of the active compounds present in the OrPh extract. This evidence is supported not only by the observation that keeping the ascidians under sterile conditions (presence of antibiotics) does not eliminate the cytotoxic effects of the extract but also by other pieces of circumstantial evidence such as i) OrPh extracts prepared from individuals collected from different geographic areas and latitudes, such as the Mediterranean Sea (Gulf of Naples and Gulf of Taranto, Italy) and Atlantic Ocean (Roscoff, France), where it is unlike the microbial communities are identical, show comparable cytotoxic activity (Russo and Gallo, 2024); ii) similar antibiotic treatments have been applied to different marine invertebrates to detect the presence of symbiotic microorganisms and their contributions to the biosynthesis of biologically active secondary metabolites (Davidson et al., 2001; Dedeine et al., 2001; Hildebrand et al., 2004); iii) examples exist in the literature of marine organisms that, after pre-treatment with antibiotics, could still biosynthesize the bioactive compounds (Hildebrand et al., 2004).

## 4 Conclusion

The first important novelty of the present work concerns the demonstration that even in a Mediterranean species of tunicates, i.e., *C. robusta*, bioactive compounds with anti-proliferative activity are present. Furthermore, since the procedure followed for the preparation of the organic extract closely resembles the one applied for the isolation of ET-743 (Ecteinascidin-743; trabectedin), one of the most clinically successful anti-tumor molecules isolated from the ascidians and approved by the FDA for clinical use under the brand name Yondelis^®^, we hypothesize that the active compounds present in the *C. robusta* organic extract may be structurally similar to ET-743. We are aware that one limitation of the present study is with regard to the absence of information on the chemical characterization of the bioactive extract. We are intensively working on the isolation and identification of the antiproliferative components, a process that is time-consuming and requires large amounts of tissue/animals, considering that the recovery yield of the bioactive compounds is extremely low (<1%).

Although at the current status of our research, it is difficult to predict the chemical nature of the bioactive compounds present in the OrPh extract, previously published data may help to orientate our future work. Apart from plitidepsin (a cyclic depsipeptide) and Ecteinascidin-743 (a tetrahydroisoquinoline alkaloid) already described in the Introduction section as two anticancer agents with important clinical outcomes, other families of ascidian-derived chemical compounds possessing biological activities are known and can be potentially present in our OrPh extract. Among these, the natural or semi-synthetic derivatives of alkaloid staurosporine, midostaurin, and lestaurtinib have been isolated from the genus *Eudistoma* sp. and have found clinical application as multi-target kinase inhibitors in leukemia (Zenkov et al., 2020). Other important agents with clinical and preclinical history include becatecarin, a chlorinated indolocarbazole isolated from different actinomycetes present in the ascidian genus *Symplegma* sp., and edotecarin, both used as topoisomerase inhibitors against multiple forms of cancer (Zenkov et al., 2020). In addition to these compounds, almost all of symbiotic origin, which have already shown promising clinical applications, many other chemical families from ascidian sources without a clinical history are currently under scrutiny. An extensive review article of these compounds has been recently published (Cooreman et al., 2023), which includes peptides, pyridoacridine alkaloids, acetylcholinesterase inhibitors, meridianins (brominated indole alkaloids), ritterazines (dimeric steroidal pyrazine alkaloids), polyandrocarpamines (2-aminoimidazolone alkaloids), lamellarins and ningalins (DOPA/TOPA-derived pyrrole-polyphenol alkaloids), mandelalides (glycosylated polyketide macrolides), iejimalides (macrolides), spiroketals (bistramides and didemnaketals), tamandarins (depsipeptides), and eudistomins (β-carboline alkaloids).

The present work investigated the “symbiotic” origin of the bioactive compounds present in the *C. robusta* extract. It is known that marine invertebrates host in their tissues a multitude of microorganisms, such as bacteria, cyanobacteria, and fungi that reside in the extra- and intracellular spaces, and in some cases, these symbiotic microorganisms can represent up to 40% of the entire biomass (Proksch et al., 2002). As discussed above, genetic analysis has highlighted that many bioactive peptides are synthesized by obligate symbiotic microorganisms not from the parent organism, such as for ET-743. For this reason, many authors have hypothesized that most bioactive compounds isolated from marine invertebrates are derived from symbiotic bacteria/microalgae or are assimilated by the animal through the food chain (Proksch et al., 2002). However, in our case, ascidians maintained “under sterility” did not show any apparent signs of functional abnormality (e.g., *in vitro* fertilization tests indicated no differences between antibiotics-treated and antibiotics-untreated groups concerning larval development). We presented data suggesting that the antibiotic treatment does not modify the extract’s anti-proliferative activity.

Finally, the most important novelty of the present work has been the demonstration that, at least in HT-29 cells, the organic extract induced cytotoxic autophagy. Ruocco et al. (2016) had discussed blueprint autophagy in 2016, so defined because it is induced and/or inhibited by marine natural products. However, in this article, none of the molecules reviewed were derived from tunicates. Therefore, to the best of our knowledge, the data reported in the present work represent one of the first evidence of autophagic activity induced by compounds derived from sea squirts.

In conclusion, we demonstrated the presence of molecules not of symbiont origin in the tissues of *C. robusta* and autophagic activity of these compounds. Therefore, *C. robusta* can be proposed as a new model for studying the mechanisms of autophagy since essential genes regulating the autophagic process have been identified in the genome of this organism (Godefroy et al., 2009). Therefore, the presence of potential compounds capable of modulating the autophagic response could not only have a possible use in tumor therapy, as for other compounds derived from marine organisms, but also perform essential physiological functions for understanding the regulation of chordate development during the evolution.

## Data Availability

The original contributions presented in the study are included in the article/Supplementary Material; further inquiries can be directed to the corresponding author.

## Footnotes

1
https://www.ncbi.nlm.nih.gov/datasets/genome/?taxon=7713
